# Targeted next-generation sequencing assays using triplet samples of normal breast tissue, primary breast cancer, and recurrent/metastatic lesions

**DOI:** 10.1186/s12885-020-07432-w

**Published:** 2020-10-01

**Authors:** Toshiaki Akahane, Naoki Kanomata, Oi Harada, Tetsumasa Yamashita, Junichi Kurebayashi, Akihide Tanimoto, Takuya Moriya

**Affiliations:** 1 Department of Pathology, Kagoshima University Graduate School of Medical and Dental Sciences, Kagoshima, Japan; 2 Department of Pathology, Hokuto Hospital, Obihiro, Hokkaido Japan; 3 Department of Pathology, St. Luke’s International Hospital, Akashi-cho 9-1, Chuoku, Tokyo, 104-8560 Japan; 4 Department of Pathology, Kawasaki Medical School, Kurashiki, Japan; 5 Department of Breast and Thyroid Surgery, Kawasaki Medical School, Kurashiki, Japan

**Keywords:** Breast cancer, Metastasis, Recurrence, Next-generation sequencing

## Abstract

**Background:**

Next-generation sequencing (NGS) has shown that recurrent/metastatic breast cancer lesions may have additional genetic changes compared with the primary tumor. These additional changes may be related to tumor progression and/or drug resistance. However, breast cancer-targeted NGS is not still widely used in clinical practice to compare the genomic profiles of primary breast cancer and recurrent/metastatic lesions.

**Methods:**

Triplet samples of genomic DNA were extracted from each patient’s normal breast tissue, primary breast cancer, and recurrent/metastatic lesion(s). A DNA library was constructed using the QIAseq Human Breast Cancer Panel (93 genes, Qiagen) and then sequenced using MiSeq (Illumina). The Qiagen web portal was utilized for data analysis.

**Results:**

Successful results for three or four samples (normal breast tissue, primary tumor, and at least one metastatic/recurrent lesion) were obtained for 11 of 35 breast cancer patients with recurrence/metastases (36 samples). We detected shared somatic mutations in all but one patient, who had a germline mutation in *TP53*. Additional mutations that were detected in recurrent/metastatic lesions compared with primary tumor were in genes including *TP53* (three patients) and one case each of *ATR*, *BLM*, *CBFB*, *EP300*, *ERBB2*, *MUC16*, *PBRM1,* and *PIK3CA*. Actionable mutations and/or copy number variations (CNVs) were detected in 73% (8/11) of recurrent/metastatic breast cancer lesions.

**Conclusions:**

The QIAseq Human Breast Cancer Panel assay showed that recurrent/metastatic breast cancers sometimes acquired additional mutations and CNV. Such additional genomic changes could provide therapeutic target.

## Background

Studies using next-generation sequencing (NGS) have demonstrated remodeling of genes in metastatic cancer compared with the primary tumor from the same individual, possibly as a result of subclonal diversification or mutational evolution [1–10]. Whole-exon or whole-genome analyses can yield valuable information but are costly and time consuming. Whole-exon sequencing analysis of metastatic breast cancers showed drug-targetable mutations in genes such as *ERBB4*, *NOTCH3*, and *ALK* [11]. A targeted NGS assay of metastatic breast cancers and their associated primary tumor using a general cancer panel can also detect drug-targetable mutations. Vasan et al. reported that 84% (43/51) of metastatic breast cancers showed at least one genomic alteration that could be targeted by currently available drugs [12]. In 2016, Muller et al. reported that 45% (10/22) of metastatic breast cancers contained molecular targets for currently available therapies, including for off-label use [13]. In these studies, comprehensive cancer panels that were not breast cancer specific were used. To our knowledge, there has been no study using breast cancer-targeted NGS of paired samples of primary breast cancer and recurrent/metastatic cancer.

The aim of the present study was to perform breast cancer-targeted NGS to compare gene mutations and copy number variations (CNVs) in samples of primary breast cancer and recurrent/metastatic lesions from the same individuals. Becuse sequencing analyses of matched tumor and normal tissue are essential [14], we performed an NGS study using three or more samples from each patient, consisting of normal breast tissue, primary breast cancer, and recurrent/metastatic lesion(s). This study aimed to clarify the practical possibility of breast cancer-targeted NGS and improve understanding of subclonal diversification or mutational evolution of metastatic breast cancers.

## Methods

### Patients and samples

One hundred seven cases of distant metastasis or local recurrence of breast cancer were collected from the pathology archives of Kawasaki Medical School Hospital from 2010 to 2017. The microscopic evaluation of these cases found that normal breast tissue, sufficient (20% or more tumor content) primary breast cancer tissue, and tissue from at least one relapse site were available for 35 patients. For 66 patients, the tumor content of samples from primary tumors and/or recurrent/metastatic tumors was less than 20%. For six patients, the primary breast cancers were resected in other institutions, so histologic specimens were not available (Supplementary Fig. 1). The protocol of the present study was approved by the Ethics Committee of Kawasaki Medical School and Hospital (approval number: 2695).

### DNA extraction and quality assessment

Formalin-fixed paraffin-embedded (FFPE) tissue blocks were obtained from the Department of Pathology at Kawasaki Medical School Hospital. DNA was extracted from the tumor and normal tissue at the primary site (breast) and from sites of metastasis and/or local recurrence. Samples of pleural or pericardial effusion were collected using the collodion bag method to generate cell blocks. Four 10-μm sections were cut from each paraffin block. Maxwell 16 FFPE Tissue LEV DNA purification kits (#AS1130; Promega, Madison, WI, USA) were employed for DNA extraction. DNA was quantified using a Qubit 2.0 Fluorometer (Thermo Fisher Scientific, Waltham, MA, USA) and Qubit dsDNA BR assay kits (#Q32850; Thermo Fisher Scientific). DNA quality was assessed by calculating the QC score (https://www.qiagen.com/us/resources/download.aspx?id=aae35658-5ef2-44b2-bd02-fbe73fe7737c&lang=en). DNA amplification for library construction was performed by quantitative polymerase chain reaction (qPCR) using QIAseq DNA QuantiMIZE Assay Kits (#DNQC-100Y-R; Qiagen, Hilden, Germany) [15].

### Next-generation sequencing

The QIAseq Human Breast Cancer Panel (93 genes, DHS-001Z; Qiagen) and the GeneRead Human Comprehensive Cancer Panel (160 genes, NGHS-501X; Qiagen) were used for library construction according to the manufacturer’s instructions. The libraries were assessed using a QIAseq Library Quant Assay Kit (#QSTF-ILZ-R; Qiagen) and applied to a MiSeq sequencer (Illumina, San Diego, CA, USA). The Qiagen web portal (https://www.qiagen.com/us/shop/genes-and-pathways/data-analysis-center-overview-page/) was utilized for data analysis [16]. For alignment, GenomeBrowse (http://goldenhelix.com/products/GenomeBrowse/index.html) was used, and GRCH37 was used as the human genome reference. A commercial bioinformatic analysis service (Mitsubishi Space Software Co. Ltd., Tokyo, Japan) was asked to interpret the GeneRead Human Comprehensive Cancer Panel results. CNV was calculated using the cloud analysis pipeline of the Qiagen web portal, and corrected for the percent tumor content. Four or more and one or fewer CNVs were regarded as significant.

### Statistical analyses

Statistical analyses were performed using IBM SPSS Statistics for Windows (v 25; IBM Corp., Armonk, NY, USA) and *P* < 0.05 was considered significant.

## Results

### Clinicopathological findings

For the 11 of the total 107 patients who were successfully analyzed (Supplementary Fig. 1), the median age was 52.5 years at diagnosis of breast cancer. The median time to first relapse was 11 months, and the overall survival was 39 months. All but one patient died from breast cancer. The breast cancer subtypes were six triple-negative, four luminal, and one HER2-enhanced. All patients but one had received chemotherapy and/or hormonal therapy (Table 1, Supplementary Table 1).

**Table 1:** Clinicopathological factors of the breast cancer patients with recurrent/metastatic lesions

Case ID	Primary or relapse	Age	Primary systemic therapy	Adjuvant therapy	Histology	pT	pN	Grade	Ly	V	ER (%)	PgR (%)	HER2	Ki67 (%)	Follow-up (months)	Outcome
K18	breast primary	41	CPA, EPI, DTX, 5’DFUR	X	ICNOS	1a	0	2	0	1	0	0	0	76.0	23	DOD
	local recurrence	42									< 1	0	1+	70.5		
K20	breast primary	37	CPA, EPI	PTX, TRA, TAM	ICNOS	2	2a	3	3	1	60	0	3+	57.1	60	DOD
	pleural effusion	42									0	0	2 + (FISH +)	45.0		
K21	breast primary	66	CPA, EPI, EXE	X, EXE	ICNOS	4b	1a	2	1	2	90	10	1+	39.5	63	DOD
	local recurrence	68									100	70	1+	22.3		
	local recurrence	69									100	90	1+	47.6		
K22	breast primary	63	no	TAM	ILC	1b	0	1	0	0	30	0	0	9.4	25	DOD
	pleural effusion	65									0	< 1	0	24.8		
K23	breast primary	65	no	no	ICNOS	1a	0	1	0	0	95	80	1+	11.8	92	LND
	local recurrence	68									95	90	1+	5.8		
K25	breast primary	35	CPA, EPI, DTX, 5’DFUR	GEM	ICNOS	2	1a	3	1	1	0	0	0	55.4	21	DOD
	local recurrence	36									0	0	1+	83.0		
K27	breast primary	55	CPA, EPI	X	ICNOS	3	0	3	3	0	5	2	1+	68.2	7	DOD
	local recurrence	55									0	0	0	87.0		
K28	breast primary	50	CPA, EPI, DTX, 5’DFUR	CPA, EPI, X	ICNOS	3	1a	3	3	2	0	0	1+	86.8	50	DOD
	local recurrence	51									0	0	1+	57.4		
K30	breast primary	68	no	5FU, CPA, EPI	ICNOS	1c	3b	3	1	0	0	0	0	80.2	12	DOD
	pleural effusion	68									0	0	0	87.0		
	pleural effusion #2	69									0	0	0	84.0		
K31	breast primary	47	CPA, EPI	CPA, X	ICNOS	1a	3a	3	1	0	0	0	0	65.4	28	DOD
	pericardial effusion	48									0	0	0	36.6		
	pleural effusion	49									0	0	0	58.4		
K32	breast primary	38	no	CPA, EPI, TAM	ICNOS	1c	1mi	2	2	1	80	2	1+	37.5	83	DOD
	lung metastasis	45									10	0	0	58.4		

*CPA* cyclophosphamide, *EPI* epirubicin, *DT* docetaxel, *5’DFUR* doxifluridine, *5FU* 5 fluorouracil, *TAM* tamoxifen, *X* capecitabine, *PTX* paclitaxel, *TRA* trastuzumab,

*EXE* exemestane, *GEM* gemcitabine, *ICNOS* invasive carcinoma of no special type, *ILC* invasive lobular carcinoma, *na* not available, *DOD* dead of disease, *LND* living without disease

### DNA quality and QIAseq human breast Cancer panel

**Fig. 1:**
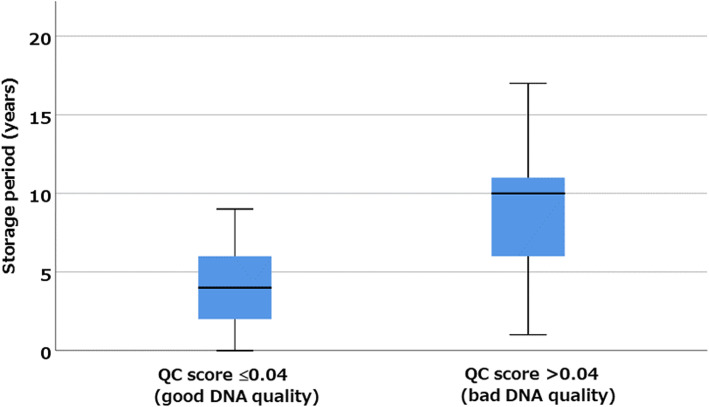
The samples with good QC scores (≤0.04) were collected more recently than those with poor QC scores (> 0.04) (*P* < 0.0005, Mann–Whitney U test). A QC score threshold of 0.04 is recommended by Qiagen

The data for DNA quality are shown in Supplementary Table 2. The QC score was calculated as the difference between the CT values obtained by qPCR using the 100-bp and 200-bp primers of the QIAseq DNA QuantiMIZE assay kit. A high QC score indicates severe DNA fragmentation. A favorable QC score (≤0.04) was obtained from 43 of 85 (50.6%) samples. The samples with good DNA quality were collected significantly more recently than those with poor DNA quality (*p* < 0.0005, Fig. 1). The FFPE blocks used in this study were 6 months to 17 years old; 66.7% (38/57) of the blocks that were ≥ 5 years old had a QC score > 0.04. We attempted to construct libraries from 14 high-QC-score samples; however, the reading depth for the molecular-tagged sites in eight cases (57.2%) was < 10, and failed to identify a mutation. The depth of molecular-tagged sites and all examined sites were both significantly correlated with the storage years (*P* < 0.0005 and r = − 0.586, *P* = 0.004 and r = − 0.412, Spearman’s correlation).

**Fig. 2:**
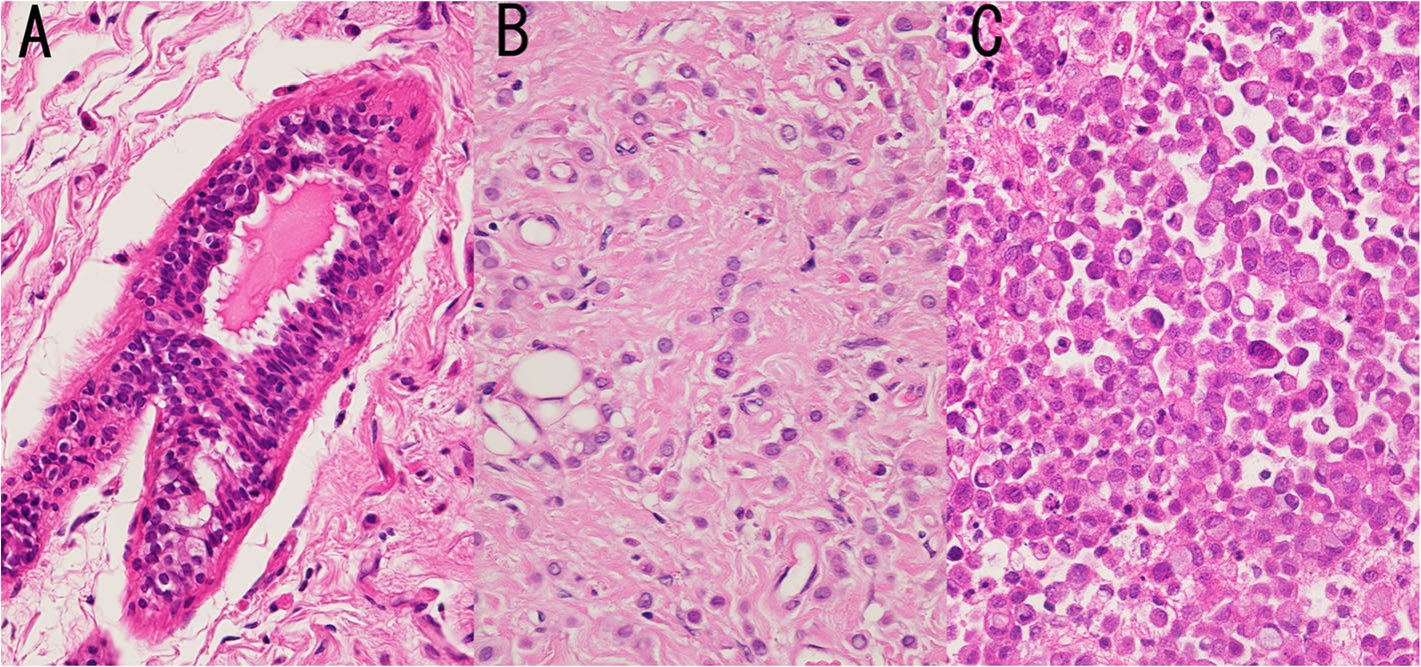
Case K22. **a** Normal breast tissue, **b** primary breast invasive lobular carcinoma showing loose trabecular growth with *CDH1* p.Q264*mutation, and **c** pleural effusion containing metastatic cancer cells with *CDH1* p.Q264*, *ERBB2* p.S310F, *CBFB* p.X27_splice and *TP53* p.R248Q mutations

Successful results were obtained from samples of 11 patients that comprised normal breast tissue, primary tumor, and at least one metastatic/recurrent lesion (36 samples, Table 1), using the QIAseq Human Breast Cancer Panel. We detected somatic driver mutations in all but one case (91%) (Table 2). The mutations shared between the primary tumor and a recurrent/metastatic lesion occurred in *TP53* (five cases), *PIK3CA* (three cases), *CDH1* (one case), *ESR1* (one case), *GATA3* (one case), and *PTEN* (one case). In five cases (45.4%), additional mutations were detected in the recurrent/metastatic lesions compared with the primary tumor. These additional mutations occurred in *TP53* in three cases and in *ATR*, *BLM*, *CBFB*, *EP300*, *ERBB2*, *MUC16*, *PBRM1*, and *PIK3CA* in one case each (Table 2, Fig. 2). No additional mutation was found in one long-surviving patient (K23) at 92 months after partial mastectomy.

**Table 2:**
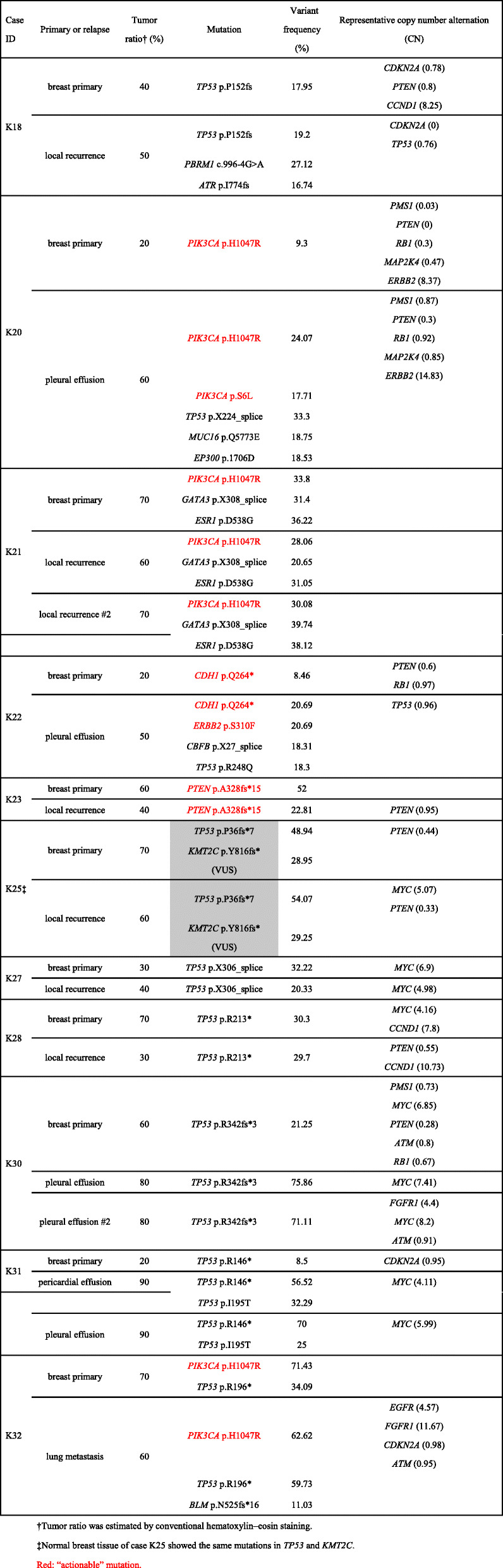
Mutations and copy number variations of primary breast cancers and recurrent/metastatic lesions

**Fig. 3:**
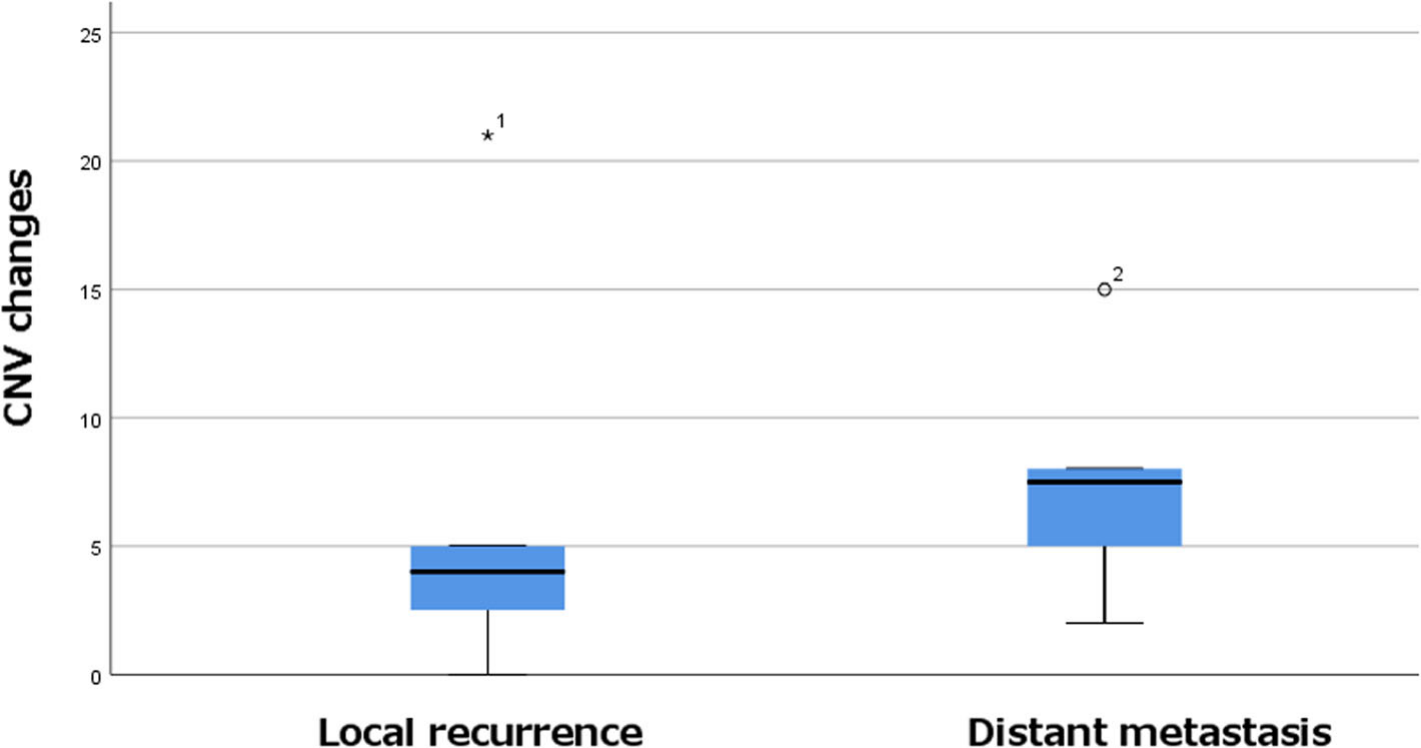
CNV seems to be higher in distant metastasis than in local recurrence, but the difference was not significant (*P* = 0.091, Mann–Whitney U test)

The results for CNV are shown in Table 2 and Supplementary Table 3. CNV appears to be higher in distant metastasis than in local recurrence, but this difference was not significant (*P* = 0.091, Fig. 3).

### GeneRead human comprehensive Cancer panel

**Fig. 4:**
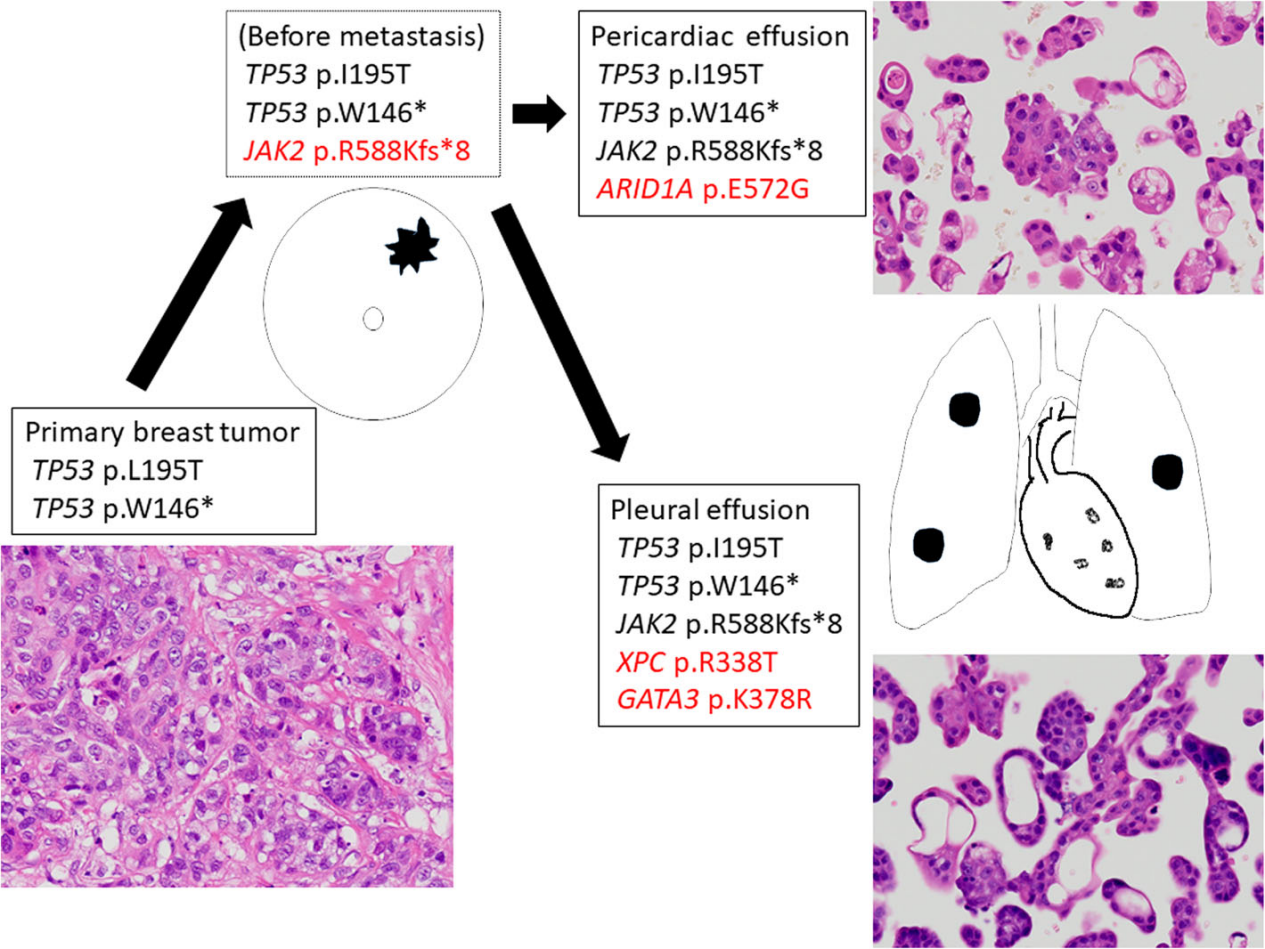
GeneRead Human Comprehensive Cancer Panel analysis for patient K31. The primary and metastatic cancer cells have similar morphology, but the pericardial and pleural disseminations contained different mutations

For patient K31, only two mutations were identified by the QIAseq Human Breast Cancer Panel. We suspected the presence of undetected mutations, so performed additional analyses using the GeneRead Human Comprehensive Cancer Panel and a commercial bioinformatic analysis service (Mitsubishi Space Software Co. Ltd). These analyses revealed an additional *JAK2* mutation (p.R588Kfs*8) that occurred before metastasis, an *ARID1A* mutation (p.E672G) that appeared in the first metastasis (pericardial fluid), and additional mutations in *XPC* (p.R338T) and *GATA3* (p.K378R) that were present in the second metastasis (pleural fluid) (Fig. 4). Although *GATA3* is included in the QIAseq Human Breast Cancer Panel, we were unable to detect this mutation by web-portal analysis because of its low variant frequency.

For patient K25, only a germ line *TP53* mutation was detected by the QIAseq Human Breast Cancer Panel. The GeneRead Human Comprehensive Cancer Panel analysis confirmed this *TP53* germ line mutation without identifying other significant mutations.

## Discussion

We showed that older FFPE materials often had lower DNA quality and could not be analyzed. The depth discrepancy between molecular-tagged sites and all sites is interesting. The Spearman’s correlation coefficients with age were − 0.586 for tagged sites and − 0.412 for all sites, suggesting that the molecular tag might be sensitive to age-related DNA damage. PAXgene tissue fixation and/or low-temperature paraffin block storing at 4 °C or − 20 °C could improve DNA quality [17].

For 66 of 107 cases (61.7%), the tumor content in samples of primary tumors and/or recurrent/metastatic tumors was less than 20%. The cell blocks of fluid materials frequently included many inflammatory cells and reactive mesothelial cells together with cancer cells; hence, the extraction of tumor DNA from these cell blocks was often inefficient. A primary breast cancer containing abundant stromal lymphoplasmacytic cell infiltration or a severe desmoplastic reaction would also inhibit effective cancer genome recovery. Thus, the development of a more efficient microdissection system is desirable.

We demonstrated that targeted NGS using the QIAseq Human Breast Cancer Panel could detect the driver mutations in all cases of breast cancer examined, except for one case with a germline *TP53* mutation that did not meet the classic criteria for Li–Fraumeni syndrome [18] or the Chompret criteria [19].

The most frequently detected mutation shared between primary and metastatic lesions was in *TP53*. Breast cancer patients with a somatic *TP53* mutation have a poor prognosis [20, 21]; unfortunately, *TP53* mutations are not presently targetable. Phosphatidylinositide 3-kinase (PI3K) inhibitor could be a good therapeutic option for cases with *PIK3CA* mutations [22–24]. Alpelisib has been reported to improve the survival patients with *PIK3CA*-altered, ER-positive, HER2-negative breast cancer [25, 26]. Alpelisib is already approved by the US Food and Drug Administration. The Lotus trial showed that ipatasertib, an oral AKT inhibitor, improved the progression-free survival of breast cancer patients with *PIK3CA/AKT/PTEN* mutations [27]. IPATunity130, a pivotal randomized phase III trial evaluating ipatasertib (IPAT) + paclitaxel for *PIK3CA/AKT1/PTEN*-altered advanced triple-negative or hormone receptor-positive HER2-negative breast cancer, is ongoing (http://ascopubs.org/doi/abs/10.1200/JCO.2018.36.15_suppl.TPS1117). Breast cancers with a *PIK3CA* mutation have a good prognosis [21]. *CDH1*-mutated breast cancer cells are sensitive to ROS1 tyrosine kinase inhibitors including foretinib or crizotinib [28]. *ESR1* mutations of breast cancer are often reported after aromatase inhibitor and/or tamoxifen therapy [29]. The SoFEA (Study of Faslodex Versus Exemestane With or Without Arimidex) trial showed that cases with *ESR1* mutations had better survival when treated with fulvestrant compared with exemestane [30].

Of the additional mutations detected, *ERBB2* p.S310F is notable because it results in activation of HER2 without gene amplification or protein overexpression [31]. A tumor with this mutation is likely to be sensitive to neratinib, as are those with G660D, R678Q, E693K, and Q709 mutations [32]. The neratinib HER Mutation Basket Study (SUMMIT) has already started [33]. A *PBRM1* mutation may evoke immunotherapy resistance [34].

Cases with PTEN loss could be treated with *PI3K/AKT* inhibitor [35]. Case K18 and K28 (triple-negative cancers) with *CCND1* gene amplification might be sensitive to CDK4/6 inhibitors [36]. Meanwhile, erdafitinib and cetuximab might be effective for cases K30 and K32 in which the metastatic lesions contained *FGFR* and *EGFR* amplifications [37, 38]. Case K30 and K32, which had decreased *ATM* might be sensitive to topotecan or the poly-(ADP-ribose) polymerase inhibitor olaparib [39]. *CDKN2A* or *RB1* downregulation could be target of palbociclib [40].

The present study with only 93 genes analyzed showed actionable mutations or CNVs in 73% (8/11) of recurrent/metastatic breast cancer lesions. This is comparable to the findings of previous studies including MSK-IMPACT (61%), the study by Vasan et al. (84%), and the study by Muller et al. (45%) [12, 13, 41].

The major limitations of the present study are its small scale and subtype bias, for which problems with DNA availability are responsible. Patients with luminal breast cancer often show late recurrence/metastasis, such as up to 18 years after diagnosis in our series. The primary tumor blocks of such cases are too old and rarely maintain sufficient DNA quality. In contrast, triple-negative breast cancer usually recurs shortly after surgery. Another limitation is the determination of the cutoff level for CNV. The relationship between drug sensitivity and CNV remains to be elucidated. Moreover, additional immunohistochemistry might be helpful for cases with altered CNVs of *EGFR* or *FGFR*. We could detect the somatic driver mutations or germline mutations in five triple-negative cancers; however, the genes covered by the QIAseq Human Breast Cancer Panel might be inadequate for analysis of triple-negative breast cancers because these cancers are known to have highly variable mutations [42].

## Conclusion

Our targeted NGS assay using the QIAseq Human Breast Cancer Panel showed that recurrent/metastatic breast cancer lesions sometimes gained additional mutation and CNV. This method assists the identification of drug-targetable mutations and changes in CNV levels. The performance of an expanded study including an analysis of drug sensitivity is to be encouraged.

## Supplementary information

**Additional file 1: Supplementary Figure 1.** Case inclusion and exclusion status.

**Additional file 2: Supplementary Table 1.** Clinicopathological factors of the breast cancer patients with recurrent/metastatic lesions.

**Additional file 3: Supplementary Table 2.** DNA quality, quantity and NGS depth of FFPE samples.

**Additional file 4: Supplementary Table 3.** Copy number variations of primary breast tumor and recurrent/metastatic lesions.

## Data Availability

The datasets generated and/or analysed during the current study are available in the SYNAPSE repository, https://www.synapse.org/#!Synapse:syn22364283/files/.

## Abbreviations

CNV: Copy number variation
CPA: Cyclophosphamide
DM: Distant metastasis
DOD: Dead of disease
DTX: Docetaxel
EPI: Epirubicin
EXE: Exemestane
FFPE: Formalin-fixed paraffin-embedded
GEM: Gemcitabine
ICNOS: Invasive carcinoma, NOS
ILC: Invasive lobular carcinoma
LND: Live without disease
LR: Local recurrence
na: Not available
NGS: Next-generation sequencing
PB: Primary breast tumor
PTX: Paclitaxel
TAM: Tamoxifen
TRA: Trastuzumab
X: Capecitabine,
5’DFUR: Doxifluridine

## References

[1] Ding L, Ellis MJ, Li S, Larson DE, Chen K, Wallis JW, Harris CC, McLellan MD, Fulton RS, Fulton LL (2010). Genome remodelling in a basal-like breast cancer metastasis and xenograft. Nature..

[2] De Mattos-Arruda L, Weigelt B, Cortes J, Won HH, Ng CK, Nuciforo P, Bidard FC, Aura C, Saura C, Peg V (2014). Capturing intra-tumor genetic heterogeneity by de novo mutation profiling of circulating cell-free tumor DNA: a proof-of-principle. Ann Oncol.

[3] Brastianos PK, Carter SL, Santagata S, Cahill DP, Taylor-Weiner A, Jones RT, Van Allen EM, Lawrence MS, Horowitz PM, Cibulskis K (2015). Genomic characterization of brain metastases reveals branched evolution and potential therapeutic targets. Cancer Discov.

[4] Juric D, Castel P, Griffith M, Griffith OL, Won HH, Ellis H, Ebbesen SH, Ainscough BJ, Ramu A, Iyer G (2015). Convergent loss of PTEN leads to clinical resistance to a PI(3)Kalpha inhibitor. Nature..

[5] Yates LR, Gerstung M, Knappskog S, Desmedt C, Gundem G, Van Loo P, Aas T, Alexandrov LB, Larsimont D, Davies H (2015). Subclonal diversification of primary breast cancer revealed by multiregion sequencing. Nat Med.

[6] Hoadley KA, Siegel MB, Kanchi KL, Miller CA, Ding L, Zhao W, He X, Parker JS, Wendl MC, Fulton RS (2016). Tumor evolution in two patients with basal-like breast Cancer: a retrospective genomics study of multiple metastases. PLoS Med.

[7] Savas P, Teo ZL, Lefevre C, Flensburg C, Caramia F, Alsop K, Mansour M, Francis PA, Thorne HA, Silva MJ (2016). The subclonal architecture of metastatic breast Cancer: results from a prospective community-based rapid autopsy program "CASCADE". PLoS Med.

[8] Yates LR, Knappskog S, Wedge D, Farmery JHR, Gonzalez S, Martincorena I, Alexandrov LB, Van Loo P, Haugland HK, Lilleng PK (2017). Genomic evolution of breast Cancer metastasis and relapse. Cancer Cell.

[9] Siegel MB, He X, Hoadley KA, Hoyle A, Pearce JB, Garrett AL, Kumar S, Moylan VJ, Brady CM, Van Swearingen AE (2018). Integrated RNA and DNA sequencing reveals early drivers of metastatic breast cancer. J Clin Invest.

[10] Bertucci F, Ng CKY, Patsouris A, Droin N, Piscuoglio S, Carbuccia N, Soria JC, Dien AT, Adnani Y, Kamal M (2019). Genomic characterization of metastatic breast cancers. Nature..

[11] Lefebvre C, Bachelot T, Filleron T, Pedrero M, Campone M, Soria JC, Massard C, Levy C, Arnedos M, Lacroix-Triki M (2016). Mutational profile of metastatic breast cancers: a retrospective analysis. PLoS Med.

[12] Vasan N, Yelensky R, Wang K, Moulder S, Dzimitrowicz H, Avritscher R, Wang B, Wu Y, Cronin MT, Palmer G (2014). A targeted next-generation sequencing assay detects a high frequency of therapeutically targetable alterations in primary and metastatic breast cancers: implications for clinical practice. Oncologist..

[13] Muller KE, Marotti JD, de Abreu FB, Peterson JD, Miller TW, Chamberlin MD, Tsongalis GJ, Tafe LJ (2016). Targeted next-generation sequencing detects a high frequency of potentially actionable mutations in metastatic breast cancers. Exp Mol Pathol.

[14] Jones S, Anagnostou V, Lytle K, Parpart-Li S, Nesselbush M, Riley DR, Shukla M, Chesnick B, Kadan M, Papp E (2015). Personalized genomic analyses for cancer mutation discovery and interpretation. Sci Transl Med.

[15] Akahane T, Yamaguchi T, Kato Y, Yokoyama S, Hamada T, Nishida Y, Higashi M, Nishihara H, Suzuki S, Ueno S (2019). Comprehensive validation of liquid-based cytology specimens for next-generation sequencing in cancer genome analysis. PLoS One.

[16] Bandoh N, Akahane T, Goto T, Kono M, Ichikawa H, Sawada T, Yamaguchi T, Nakano H, Kawase Y, Kato Y (2018). Targeted next-generation sequencing of cancer-related genes in thyroid carcinoma: a single institution's experience. Oncol Lett.

[17] Groelz D, Viertler C, Pabst D, Dettmann N, Zatloukal K. Impact of storage conditions on the quality of nucleic acids in paraffin embedded tissues. PLoS One. 2018;13(9):e0203608. 10.1371/journal.pone.0203608 PMC6128582 30192857

[18] Li FP, Fraumeni JF, Mulvihill JJ, Blattner WA, Dreyfus MG, Tucker MA, Miller RW (1988). A cancer family syndrome in twenty-four kindreds. Cancer Res.

[19] Chompret A, Abel A, Stoppa-Lyonnet D, Brugieres L, Pages S, Feunteun J, Bonaiti-Pellie C (2001). Sensitivity and predictive value of criteria for p53 germline mutation screening. J Med Genet.

[20] Silwal-Pandit L, Vollan HK, Chin SF, Rueda OM, McKinney S, Osako T, Quigley DA, Kristensen VN, Aparicio S, Borresen-Dale AL (2014). TP53 mutation spectrum in breast cancer is subtype specific and has distinct prognostic relevance. Clin Cancer Res.

[21] Griffith OL, Spies NC, Anurag M, Griffith M, Luo J, Tu D, Yeo B, Kunisaki J, Miller CA, Krysiak K (2018). The prognostic effects of somatic mutations in ER-positive breast cancer. Nat Commun.

[22] Baselga J, Im SA, Iwata H, Cortes J, De Laurentiis M, Jiang Z, Arteaga CL, Jonat W, Clemons M, Ito Y (2017). Buparlisib plus fulvestrant versus placebo plus fulvestrant in postmenopausal, hormone receptor-positive, HER2-negative, advanced breast cancer (BELLE-2): a randomised, double-blind, placebo-controlled, phase 3 trial. Lancet Oncol..

[23] Di Leo A, Johnston S, Lee KS, Ciruelos E, Lonning PE, Janni W, O'Regan R, Mouret-Reynier MA, Kalev D, Egle D (2018). Buparlisib plus fulvestrant in postmenopausal women with hormone-receptor-positive, HER2-negative, advanced breast cancer progressing on or after mTOR inhibition (BELLE-3): a randomised, double-blind, placebo-controlled, phase 3 trial. Lancet Oncol..

[24] Tamura K, Kodaira M, Shimizu C, Yonemori K, Yunokawa M, Shimomura A, Kobayashi T, Nakano K, Tomomatsu J, Ito Y (2018). Phase I study of taselisib in Japanese patients with advanced solid tumors or hormone receptor-positive advanced breast cancer. Cancer Sci.

[25] Andre F, Ciruelos E, Rubovszky G, Campone M, Loibl S, Rugo HS, Iwata H, Conte P, Mayer IA, Kaufman B (2019). Alpelisib for PIK3CA-mutated, hormone receptor-positive advanced breast Cancer. N Engl J Med.

[26] Juric D, Janku F, Rodon J, Burris HA, Mayer IA, Schuler M, Seggewiss-Bernhardt R, Gil-Martin M, Middleton MR, Baselga J (2019). Alpelisib plus Fulvestrant in PIK3CA-altered and PIK3CA-wild-type estrogen receptor-positive advanced breast Cancer: a phase 1b clinical trial. JAMA Oncol.

[27] Kim SB, Dent R, Im SA, Espie M, Blau S, Tan AR, Isakoff SJ, Oliveira M, Saura C, Wongchenko MJ (2017). Ipatasertib plus paclitaxel versus placebo plus paclitaxel as first-line therapy for metastatic triple-negative breast cancer (LOTUS): a multicentre, randomised, double-blind, placebo-controlled, phase 2 trial. Lancet Oncol.

[28] Bajrami I, Marlow R, van de Ven M, Brough R, Pemberton HN, Frankum J, Song F, Rafiq R, Konde A, Krastev DB (2018). E-cadherin/ROS1 inhibitor synthetic lethality in breast Cancer. Cancer Discov..

[29] Zundelevich A, Dadiani M, Kahana-Edwin S, Itay A, Sella T, Gadot M, Cesarkas K, Farage-Barhom S, Saar EG, Eyal E (2020). ESR1 mutations are frequent in newly diagnosed metastatic and loco-regional recurrence of endocrine-treated breast cancer and carry worse prognosis. Breast Cancer Res.

[30] Fribbens C, O'Leary B, Kilburn L, Hrebien S, Garcia-Murillas I, Beaney M, Cristofanilli M, Andre F, Loi S, Loibl S (2016). Plasma ESR1 mutations and the treatment of estrogen receptor-positive advanced breast Cancer. J Clin Oncol.

[31] Greulich H, Kaplan B, Mertins P, Chen TH, Tanaka KE, Yun CH, Zhang X, Lee SH, Cho J, Ambrogio L (2012). Functional analysis of receptor tyrosine kinase mutations in lung cancer identifies oncogenic extracellular domain mutations of ERBB2. Proc Natl Acad Sci U S A.

[32] Pahuja KB, Nguyen TT, Jaiswal BS, Prabhash K, Thaker TM, Senger K, Chaudhuri S, Kljavin NM, Antony A, Phalke S (2018). Actionable activating oncogenic ERBB2/HER2 Transmembrane and Juxtamembrane domain mutations. Cancer Cell.

[33] Hyman DM, Piha-Paul SA, Won H, Rodon J, Saura C, Shapiro GI, Juric D, Quinn DI, Moreno V, Doger B (2018). HER kinase inhibition in patients with HER2- and HER3-mutant cancers. Nature..

[34] Otto G (2018). Kidney cancer: PBRM1 loss promotes tumour response to immunotherapy. Nat Rev Clin Oncol.

[35] Xing Y, Lin NU, Maurer MA, Chen H, Mahvash A, Sahin A, Akcakanat A, Li Y, Abramson V, Litton J (2019). Phase II trial of AKT inhibitor MK-2206 in patients with advanced breast cancer who have tumors with PIK3CA or AKT mutations, and/or PTEN loss/PTEN mutation. Breast Cancer Res.

[36] Hamilton E, Infante JR (2016). Targeting CDK4/6 in patients with cancer. Cancer Treat Rev.

[37] Roncato F, Rruga F, Porcu E, Casarin E, Ronca R, Maccarinelli F, Realdon N, Basso G, Alon R, Viola G (2018). Improvement and extension of anti-EGFR targeting in breast cancer therapy by integration with the Avidin-nucleic-acid-Nano-assemblies. Nat Commun.

[38] Karkera JD, Cardona GM, Bell K, Gaffney D, Portale JC, Santiago-Walker A, Moy CH, King P, Sharp M, Bahleda R (2017). Oncogenic characterization and pharmacologic sensitivity of activating fibroblast growth factor receptor (FGFR) genetic alterations to the selective FGFR inhibitor Erdafitinib. Mol Cancer Ther.

[39] Balmus G, Pilger D, Coates J, Demir M, Sczaniecka-Clift M, Barros AC, Woods M, Fu B, Yang F, Chen E (2019). ATM orchestrates the DNA-damage response to counter toxic non-homologous end-joining at broken replication forks. Nat Commun.

[40] Frisone D, Charrier M, Clement S, Christinat Y, Thouvenin L, Homicsko K, Michielin O, Bodmer A, Chappuis PO, McKee TA (2020). Durable response to palbociclib and letrozole in ovarian cancer with CDKN2A loss. Cancer Biol Ther.

[41] Zehir A, Benayed R, Shah RH, Syed A, Middha S, Kim HR, Srinivasan P, Gao J, Chakravarty D, Devlin SM (2017). Mutational landscape of metastatic cancer revealed from prospective clinical sequencing of 10,000 patients. Nat Med.

[42] Lips EH, Michaut M, Hoogstraat M, Mulder L, Besselink NJ, Koudijs MJ, Cuppen E, Voest EE, Bernards R, Nederlof PM et al. Next generation sequencing of triple negative breast cancer to find predictors for chemotherapy response. Breast Cancer Res. 2015;17(1):134. 10.1186/s13058-015-0642-8 PMC4592753 26433948

