# Supplementary figures and images for: Targeted next-generation sequencing assays using triplet samples of normal breast tissue, primary breast cancer, and recurrent/metastatic lesions
Source: BMC Cancer. 2020 Oct 1;20:944. doi: 10.1186/s12885-020-07432-w (PMC7528467; doi:10.1186/s12885-020-07432-w)

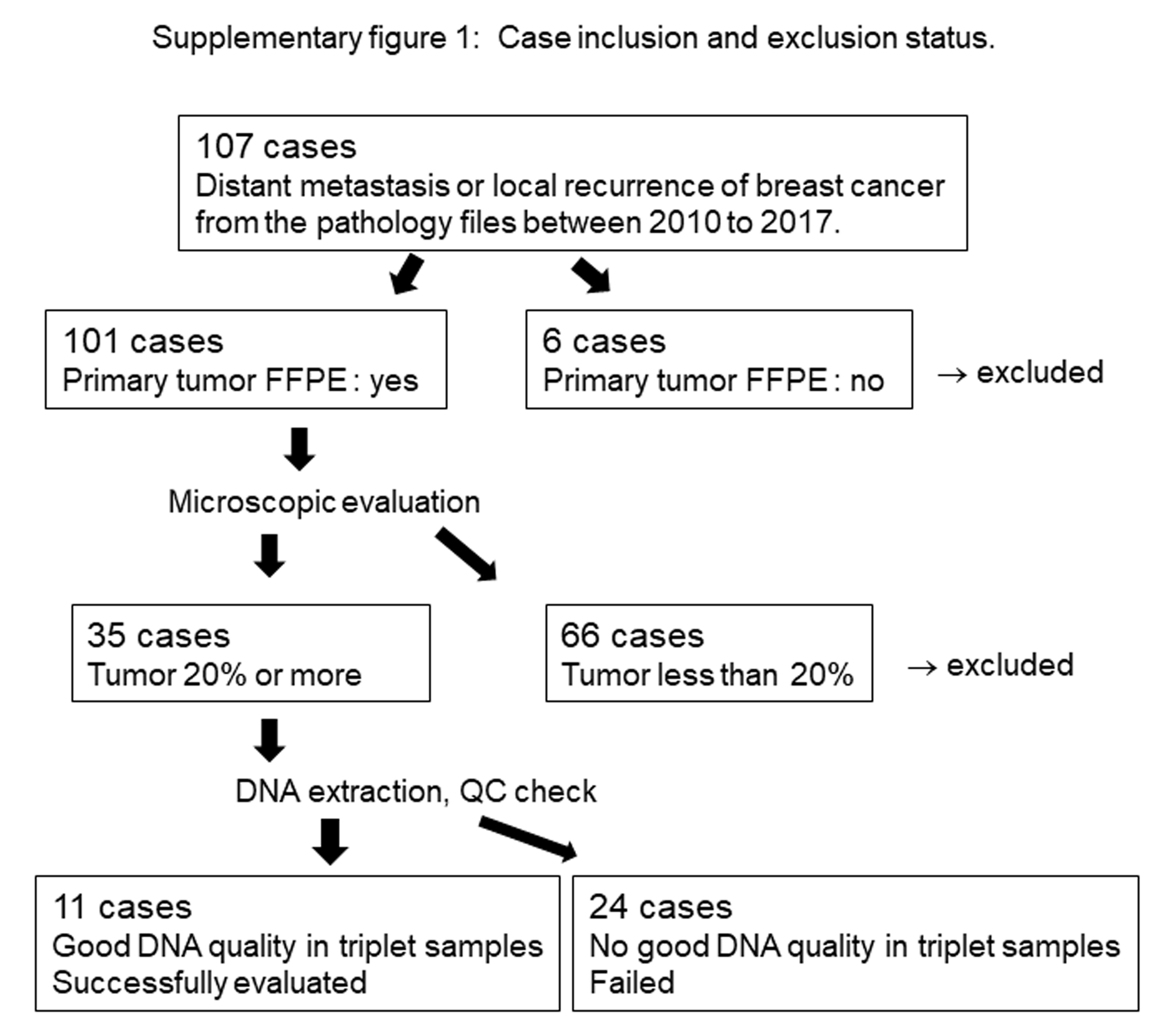

Supplement: Supplementary file 1 — Additional file 1: Supplementary Figure 1. Case inclusion and exclusion status. [file 12885_2020_7432_MOESM1_ESM.tif]
